# Lipolysis and Bacteriolysis of the Tubercle Bacillus

**Published:** 1918-11

**Authors:** 


**Lipolysis and Bacteriolysis of the Tubercle Bacillus.** E. A. Bossan and E. Le Moignic, Le Prog. Med., Mch. 23. Metchnikoff and Metalnikoff have called attention to the immunity of the honey-comb mite, which they ascribe to a specific lipase. Deycke, Much and one of the authors have shown that the envelope of the tubercle bacillus as well as of the non-pathogenic streptothrix leproe, contains a resistant wax which, injected into a tuberculous organism, produces active antibodies. They were unable to obtain satisfactory biologic results, as the attempt at extraction with hot xylene, boiling alcohol and ether decomposed too much of the bacillary contents and largely prevented a vaccinating power. Deycke, in 1906, showed that, in analogy with albuminoids and lipoids, the fatty substance extracted from acido-resistant bacilli (named nastin) could produce specific antibodies. The authors have shown that Le Moignic's oily mixture dissolves the waxes of the tubercle bacillus and that the acid resistance falls rapidly to zero in about 3 weeks. They have employed two kinds of vaccine, a complete suspension still containing the bodies of the bacilli, and one freed from bacilli by filtration. Using the latter in guinea pigs and rabbits, lipolytic and bacteriolytic anti-bodies were produced. Controls died 3-4 weeks after injection with tubercle bacilli. The vaccinated animals survived several weeks to several months after the injection of 3 centigrams of very virulent tubercle bacilli. Cultures to which blood from vaccinated animals was added, after even 12 hours incubation at 37 deg. showed morphologic changes, with development of 2-4 very darkly staining granules connected by a lighter-stained filament. Bacilli with a single granule at one end resembled spermatozoa. Some bacilli took the couter stain. Similar appearances were found in bacilli from the vaccinated and infected animals. They imply that the obstacle to successful therapeutic use of the extract of bacillary waxes in Le Moignic's oily mixture, is that the production of lipolytic and bacteriolytic antibodies does not directly destroy the vitality of the bacilli, experimentally or accidentally introduced.

